# *cis*-Dibromidobis(2-phenyl­pyridine-κ*N*)platinum(II)

**DOI:** 10.1107/S160053681203471X

**Published:** 2012-08-11

**Authors:** Kwang Ha

**Affiliations:** a School of Applied Chemical Engineering, The Research Institute of Catalysis, Chonnam National University, Gwangju 500-757, Republic of Korea

## Abstract

In the title complex, [PtBr_2_(C_11_H_9_N)_2_], the Pt^II^ ion has a distorted *cis*-Br_2_N_2_ square-planar coordination geometry defined by two N atoms from two 2-phenyl­pyridine (ppy) ligands and two Br^−^ anions. The ppy ligands are not planar, the dihedral angles between the pyridine and benzene rings being 49.0 (3) and 47.3 (3)°. In the crystal, the complex mol­ecules are stacked in columns along the *a* axis. In the columns, there are numerous intra- and inter­molecular π–π inter­actions between the six-membered rings, the shortest ring centroid–centroid distance being 3.774 (6) Å.

## Related literature

For the crystal structures of the related Pt^II^ and Pd^II^ complexes, *cis*-[PtCl_2_(ppy)_2_] and *trans*-[Pd*X*_2_(ppy)_2_] (*X* = Cl or I), see: Yoshinari *et al.* (2010 ▶); Ha (2011 ▶, 2012 ▶).
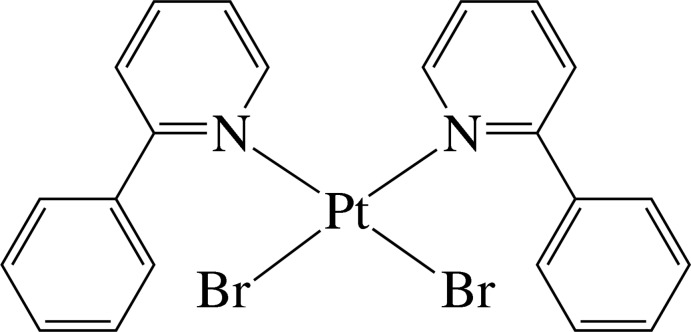


## Experimental

### 

#### Crystal data

[PtBr_2_(C_11_H_9_N)_2_]
*M* *_r_* = 665.29
Monoclinic, 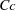

*a* = 7.6268 (9) Å
*b* = 18.277 (2) Å
*c* = 15.1626 (18) Å
β = 96.948 (2)°
*V* = 2098.1 (4) Å^3^
*Z* = 4
Mo *K*α radiation
μ = 10.51 mm^−1^
*T* = 200 K
0.24 × 0.20 × 0.14 mm

#### Data collection

Bruker SMART 1000 CCD diffractometer
Absorption correction: multi-scan (*SADABS*; Bruker, 2000 ▶) *T*_min_ = 0.729, *T*_max_ = 1.000
6126 measured reflections
2931 independent reflections
2645 reflections with *I* > 2σ(*I*)
*R*_int_ = 0.029

#### Refinement

*R*[*F* ^2^ > 2σ(*F* ^2^)] = 0.028
*wR*(*F* ^2^) = 0.068
*S* = 1.04
2931 reflections
244 parameters
2 restraints
H-atom parameters constrained
Δρ_max_ = 1.73 e Å^−3^
Δρ_min_ = −0.93 e Å^−3^
Absolute structure: Flack (1983 ▶), 856 Friedel pairs
Flack parameter: −0.037 (13)

### 

Data collection: *SMART* (Bruker, 2000 ▶); cell refinement: *SAINT* (Bruker, 2000 ▶); data reduction: *SAINT*; program(s) used to solve structure: *SHELXS97* (Sheldrick, 2008 ▶); program(s) used to refine structure: *SHELXL97* (Sheldrick, 2008 ▶); molecular graphics: *ORTEP-3* (Farrugia, 1997 ▶) and *PLATON* (Spek, 2009 ▶); software used to prepare material for publication: *SHELXL97*.

## Supplementary Material

Crystal structure: contains datablock(s) global. DOI: 10.1107/S160053681203471X/xu5600sup1.cif

Additional supplementary materials:  crystallographic information; 3D view; checkCIF report

## Figures and Tables

**Table 1:** Selected bond lengths (Å)

Pt1—N1	2.075 (8)
Pt1—N2	2.074 (10)
Pt1—Br1	2.4216 (11)
Pt1—Br2	2.4258 (15)

**Fig. 1.**
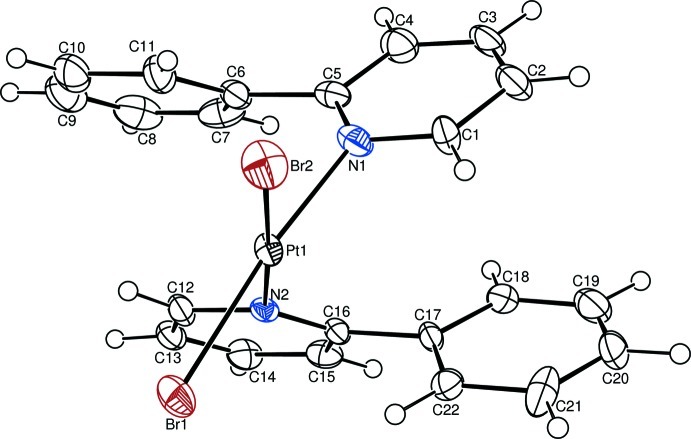
A view of the molecular structure of the title complex, with displacement ellipsoids drawn at the 40% probability level and the atom numbering.

**Fig. 2.**
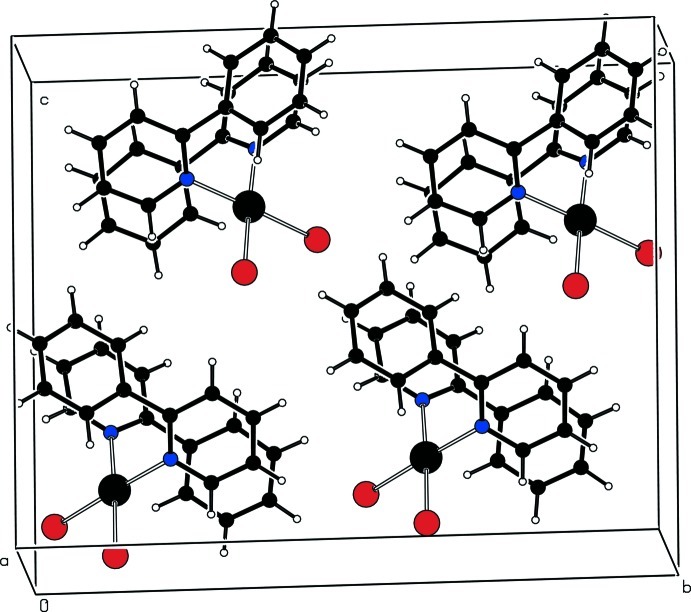
A view of the unit-cell contents of the title complex.

## supplementary crystallographic information

### Comment

Crystal structures of the related Pt^II^ and Pd^II^ complexes, such as *cis*-[PtCl_2_(ppy)_2_] (ppy = 2-phenylpyridine, C_11_H_9_N) (Yoshinari *et al.*, 2010) and *trans*-[Pd*X*_2_(ppy)_2_] (*X* = Cl or I) (Ha, 2011; Ha, 2012), have been investigated previously.

The Pt^II^ ion in the title complex, [PtBr_2_(ppy)_2_], has a distorted *cis*-Br_2_N_2_ square-planar coordination geometry defined by two N atoms from two ppy ligands and two Br^-^ anions (Fig. 1). The Pt—N and Pt—Br bond lengths are nearly equivalent, respectively (Table 1). In the crystal, the two pyridine rings are inclined to the least-squares plane of the PtBr_2_N_2_ unit [maximum deviation = 0.092 (4) Å], making dihedral angles of 61.6 (2)° and 64.0 (2)°. The ppy ligands are not planar, the dihedral angles between the pyridine and benzene rings being 49.0 (3)° and 47.3 (3)°. The complex molecules are stacked in columns along the *a* axis. In the columns, numerous intra- and intermolecular π-π interactions between the six-membered rings are present, the shortest ring centroid-centroid distance being 3.774 (6) Å (Fig. 2).

### Experimental

To a solution of K_2_PtBr_4_ (0.2391 g, 0.403 mmol) in H_2_O (20 ml)/MeOH (100 ml) was added 2-phenylpyridine (0.1810 g, 1.166 mmol) and stirred for 7 h at room temperature. The formed brown precipitate was removed by filtration and the solvent of the filtrate was evaporated. The residue was washed with H_2_O and dried at 323 K, to give a yellow powder (0.1672 g). Crystals suitable for X-ray analysis were obtained by slow evaporation from a CH_3_CN solution at room temperature.

### Refinement

H atoms were positioned geometrically and allowed to ride on their respective parent atoms: C—H = 0.95 Å and *U*_iso_(H) = 1.2*U*_eq_(C). The highest peak (1.73 e Å^-3^) and the deepest hole (-0.93 e Å^-3^) in the difference Fourier map are located 1.31 Å and 0.88 Å, respectively, from the atoms H11 and Pt1.

### Crystal data

**Table:**

[PtBr_2_(C_11_H_9_N)_2_]	*F*(000) = 1248
*M**_r_* = 665.29	*D*_x_ = 2.106 Mg m^−^^3^
Monoclinic, *C**c*	Mo *K*α radiation, λ = 0.71073 Å
Hall symbol: C -2yc	Cell parameters from 4325 reflections
*a* = 7.6268 (9) Å	θ = 2.2–26.0°
*b* = 18.277 (2) Å	µ = 10.51 mm^−^^1^
*c* = 15.1626 (18) Å	*T* = 200 K
β = 96.948 (2)°	Stick, yellow
*V* = 2098.1 (4) Å^3^	0.24 × 0.20 × 0.14 mm
*Z* = 4	

### Data collection

**Table:**

Bruker SMART 1000 CCD diffractometer	2931 independent reflections
Radiation source: fine-focus sealed tube	2645 reflections with *I* > 2σ(*I*)
Graphite monochromator	*R*_int_ = 0.029
φ and ω scans	θ_max_ = 26.0°, θ_min_ = 2.6°
Absorption correction: multi-scan (*SADABS*; Bruker, 2000)	*h* = −9→8
*T*_min_ = 0.729, *T*_max_ = 1.000	*k* = −22→22
6126 measured reflections	*l* = −18→17

### Refinement

**Table:**

Refinement on *F*^2^	Secondary atom site location: difference Fourier map
Least-squares matrix: full	Hydrogen site location: inferred from neighbouring sites
*R*[*F*^2^ > 2σ(*F*^2^)] = 0.028	H-atom parameters constrained
*wR*(*F*^2^) = 0.068	*w* = 1/[σ^2^(*F*_o_^2^) + (0.0286*P*)^2^] where *P* = (*F*_o_^2^ + 2*F*_c_^2^)/3
*S* = 1.04	(Δ/σ)_max_ = 0.001
2931 reflections	Δρ_max_ = 1.73 e Å^−^^3^
244 parameters	Δρ_min_ = −0.93 e Å^−^^3^
2 restraints	Absolute structure: Flack (1983), 856 Friedel pairs
Primary atom site location: structure-invariant direct methods	Flack parameter: −0.037 (13)

### Special details

**Table:**

Geometry. All e.s.d.'s (except the e.s.d. in the dihedral angle between two l.s. planes) are estimated using the full covariance matrix. The cell e.s.d.'s are taken into account individually in the estimation of e.s.d.'s in distances, angles and torsion angles; correlations between e.s.d.'s in cell parameters are only used when they are defined by crystal symmetry. An approximate (isotropic) treatment of cell e.s.d.'s is used for estimating e.s.d.'s involving l.s. planes.
Refinement. Refinement of *F*^2^ against ALL reflections. The weighted *R*-factor *wR* and goodness of fit *S* are based on *F*^2^, conventional *R*-factors *R* are based on *F*, with *F* set to zero for negative *F*^2^. The threshold expression of *F*^2^ > σ(*F*^2^) is used only for calculating *R*-factors(gt) *etc*. and is not relevant to the choice of reflections for refinement. *R*-factors based on *F*^2^ are statistically about twice as large as those based on *F*, and *R*- factors based on ALL data will be even larger.

### Fractional atomic coordinates and isotropic or equivalent isotropic displacement parameters (Å^2^)

**Table:**

	*x*	*y*	*z*	*U*_iso_*/*U*_eq_	
Pt1	0.39565 (2)	0.136006 (14)	0.162689 (18)	0.02282 (10)	
Br1	0.19970 (16)	0.03626 (5)	0.11234 (8)	0.0369 (3)	
Br2	0.5217 (2)	0.14016 (7)	0.02336 (11)	0.0415 (4)	
N1	0.5395 (11)	0.2278 (4)	0.2077 (6)	0.030 (2)	
N2	0.2978 (14)	0.1281 (4)	0.2842 (7)	0.021 (2)	
C1	0.4929 (14)	0.2895 (5)	0.1628 (8)	0.035 (3)	
H1	0.4181	0.2865	0.1081	0.042*	
C2	0.5505 (16)	0.3567 (5)	0.1939 (10)	0.042 (3)	
H2	0.5197	0.3997	0.1605	0.051*	
C3	0.6514 (16)	0.3604 (5)	0.2727 (10)	0.044 (3)	
H3	0.6870	0.4068	0.2970	0.052*	
C4	0.7031 (14)	0.2986 (5)	0.3180 (8)	0.040 (3)	
H4	0.7776	0.3013	0.3728	0.048*	
C5	0.6453 (13)	0.2306 (5)	0.2829 (7)	0.029 (2)	
C6	0.7103 (13)	0.1613 (5)	0.3294 (7)	0.029 (2)	
C7	0.7024 (15)	0.1529 (7)	0.4178 (8)	0.043 (3)	
H7	0.6555	0.1912	0.4503	0.052*	
C8	0.7627 (16)	0.0885 (7)	0.4618 (9)	0.052 (3)	
H8	0.7560	0.0824	0.5235	0.063*	
C9	0.8311 (17)	0.0349 (7)	0.4137 (10)	0.056 (4)	
H9	0.8734	−0.0087	0.4429	0.067*	
C10	0.8406 (15)	0.0420 (6)	0.3247 (9)	0.046 (3)	
H10	0.8896	0.0036	0.2931	0.055*	
C11	0.7788 (14)	0.1051 (5)	0.2802 (8)	0.036 (3)	
H11	0.7827	0.1102	0.2181	0.043*	
C12	0.3481 (13)	0.0673 (4)	0.3308 (7)	0.027 (2)	
H12	0.4130	0.0310	0.3039	0.032*	
C13	0.3097 (13)	0.0563 (5)	0.4142 (7)	0.029 (2)	
H13	0.3388	0.0115	0.4441	0.035*	
C14	0.2264 (13)	0.1122 (5)	0.4554 (7)	0.033 (2)	
H14	0.2045	0.1079	0.5156	0.040*	
C15	0.1768 (13)	0.1735 (6)	0.4071 (7)	0.031 (2)	
H15	0.1160	0.2115	0.4335	0.037*	
C16	0.2145 (12)	0.1813 (5)	0.3183 (6)	0.023 (2)	
C17	0.1496 (12)	0.2463 (4)	0.2664 (7)	0.024 (2)	
C18	0.1739 (13)	0.3160 (5)	0.3025 (8)	0.035 (3)	
H18	0.2335	0.3229	0.3606	0.042*	
C19	0.1086 (17)	0.3755 (6)	0.2510 (11)	0.052 (4)	
H19	0.1232	0.4236	0.2745	0.062*	
C20	0.0250 (16)	0.3656 (6)	0.1683 (10)	0.046 (3)	
H20	−0.0162	0.4068	0.1337	0.055*	
C21	−0.0010 (15)	0.2976 (6)	0.1341 (8)	0.045 (3)	
H21	−0.0622	0.2914	0.0762	0.054*	
C22	0.0612 (12)	0.2370 (5)	0.1830 (7)	0.028 (2)	
H22	0.0426	0.1892	0.1589	0.033*	

### Atomic displacement parameters (Å^2^)

**Table:**

	*U*^11^	*U*^22^	*U*^33^	*U*^12^	*U*^13^	*U*^23^
Pt1	0.02761 (18)	0.01791 (14)	0.02340 (18)	−0.0005 (2)	0.00489 (12)	0.0005 (2)
Br1	0.0466 (7)	0.0262 (5)	0.0378 (6)	−0.0076 (5)	0.0053 (5)	−0.0108 (5)
Br2	0.0546 (10)	0.0435 (8)	0.0291 (8)	−0.0010 (6)	0.0162 (7)	0.0022 (6)
N1	0.032 (5)	0.026 (4)	0.035 (5)	−0.008 (4)	0.012 (4)	−0.002 (4)
N2	0.018 (5)	0.022 (4)	0.021 (6)	−0.005 (3)	−0.003 (4)	0.006 (4)
C1	0.040 (6)	0.019 (4)	0.048 (7)	0.001 (4)	0.009 (5)	0.008 (5)
C2	0.044 (7)	0.024 (5)	0.062 (9)	−0.010 (5)	0.019 (7)	0.001 (5)
C3	0.039 (7)	0.020 (5)	0.075 (10)	−0.013 (5)	0.020 (7)	−0.010 (6)
C4	0.038 (7)	0.035 (6)	0.047 (7)	−0.006 (5)	0.008 (5)	−0.009 (5)
C5	0.024 (5)	0.031 (5)	0.033 (6)	−0.008 (4)	0.005 (4)	−0.005 (5)
C6	0.027 (6)	0.025 (4)	0.033 (6)	−0.006 (4)	0.003 (4)	0.003 (4)
C7	0.026 (6)	0.067 (8)	0.034 (7)	−0.008 (6)	−0.003 (5)	−0.011 (6)
C8	0.046 (7)	0.067 (8)	0.042 (7)	−0.020 (7)	−0.002 (6)	0.008 (7)
C9	0.046 (8)	0.046 (7)	0.071 (10)	−0.009 (6)	−0.011 (7)	0.026 (7)
C10	0.040 (7)	0.032 (6)	0.061 (9)	−0.007 (5)	−0.012 (6)	−0.002 (6)
C11	0.040 (6)	0.028 (5)	0.039 (7)	−0.001 (5)	0.002 (5)	0.008 (5)
C12	0.032 (5)	0.017 (4)	0.031 (6)	−0.004 (4)	0.000 (4)	0.001 (4)
C13	0.029 (6)	0.025 (5)	0.033 (6)	−0.005 (4)	0.001 (5)	0.007 (4)
C14	0.033 (6)	0.041 (5)	0.026 (6)	−0.006 (5)	0.005 (5)	0.013 (5)
C15	0.027 (6)	0.037 (5)	0.029 (6)	−0.007 (4)	0.010 (4)	−0.007 (5)
C16	0.022 (5)	0.018 (4)	0.027 (5)	−0.004 (4)	0.001 (4)	0.004 (4)
C17	0.026 (5)	0.014 (4)	0.034 (6)	0.000 (4)	0.013 (4)	0.002 (4)
C18	0.026 (5)	0.026 (5)	0.053 (7)	−0.002 (4)	0.005 (5)	−0.002 (5)
C19	0.039 (7)	0.026 (5)	0.095 (12)	−0.005 (5)	0.026 (8)	−0.006 (7)
C20	0.038 (7)	0.027 (6)	0.076 (10)	0.003 (5)	0.018 (7)	0.021 (6)
C21	0.049 (7)	0.044 (7)	0.044 (7)	0.023 (6)	0.012 (6)	0.013 (6)
C22	0.024 (5)	0.029 (5)	0.030 (6)	0.002 (4)	0.005 (4)	0.001 (4)

### Geometric parameters (Å, º)

**Table:**

Pt1—N1	2.075 (8)	C9—H9	0.9500
Pt1—N2	2.074 (10)	C10—C11	1.390 (14)
Pt1—Br1	2.4216 (11)	C10—H10	0.9500
Pt1—Br2	2.4258 (15)	C11—H11	0.9500
N1—C5	1.316 (13)	C12—C13	1.347 (14)
N1—C1	1.343 (12)	C12—H12	0.9500
N2—C16	1.303 (12)	C13—C14	1.391 (14)
N2—C12	1.346 (12)	C13—H13	0.9500
C1—C2	1.368 (14)	C14—C15	1.366 (14)
C1—H1	0.9500	C14—H14	0.9500
C2—C3	1.343 (19)	C15—C16	1.418 (13)
C2—H2	0.9500	C15—H15	0.9500
C3—C4	1.356 (16)	C16—C17	1.477 (12)
C3—H3	0.9500	C17—C22	1.370 (14)
C4—C5	1.400 (13)	C17—C18	1.389 (13)
C4—H4	0.9500	C18—C19	1.396 (17)
C5—C6	1.505 (13)	C18—H18	0.9500
C6—C7	1.358 (16)	C19—C20	1.348 (19)
C6—C11	1.406 (14)	C19—H19	0.9500
C7—C8	1.402 (17)	C20—C21	1.351 (16)
C7—H7	0.9500	C20—H20	0.9500
C8—C9	1.363 (17)	C21—C22	1.385 (13)
C8—H8	0.9500	C21—H21	0.9500
C9—C10	1.366 (18)	C22—H22	0.9500
			
N2—Pt1—N1	89.8 (3)	C9—C10—C11	120.1 (12)
N2—Pt1—Br1	87.3 (3)	C9—C10—H10	119.9
N1—Pt1—Br1	173.9 (2)	C11—C10—H10	119.9
N2—Pt1—Br2	176.9 (3)	C10—C11—C6	118.4 (10)
N1—Pt1—Br2	90.7 (2)	C10—C11—H11	120.8
Br1—Pt1—Br2	92.50 (5)	C6—C11—H11	120.8
C5—N1—C1	120.2 (9)	N2—C12—C13	122.4 (10)
C5—N1—Pt1	124.1 (7)	N2—C12—H12	118.8
C1—N1—Pt1	114.5 (7)	C13—C12—H12	118.8
C16—N2—C12	121.9 (10)	C12—C13—C14	118.4 (9)
C16—N2—Pt1	123.1 (7)	C12—C13—H13	120.8
C12—N2—Pt1	114.4 (7)	C14—C13—H13	120.8
N1—C1—C2	121.6 (12)	C15—C14—C13	118.2 (9)
N1—C1—H1	119.2	C15—C14—H14	120.9
C2—C1—H1	119.2	C13—C14—H14	120.9
C3—C2—C1	118.6 (11)	C14—C15—C16	121.1 (9)
C3—C2—H2	120.7	C14—C15—H15	119.4
C1—C2—H2	120.7	C16—C15—H15	119.4
C2—C3—C4	120.5 (10)	N2—C16—C15	117.8 (9)
C2—C3—H3	119.7	N2—C16—C17	122.5 (9)
C4—C3—H3	119.7	C15—C16—C17	119.5 (8)
C3—C4—C5	119.2 (11)	C22—C17—C18	120.3 (9)
C3—C4—H4	120.4	C22—C17—C16	119.1 (8)
C5—C4—H4	120.4	C18—C17—C16	120.5 (9)
N1—C5—C4	119.7 (10)	C17—C18—C19	118.2 (11)
N1—C5—C6	120.4 (9)	C17—C18—H18	120.9
C4—C5—C6	119.9 (9)	C19—C18—H18	120.9
C7—C6—C11	120.4 (10)	C20—C19—C18	120.8 (11)
C7—C6—C5	120.3 (9)	C20—C19—H19	119.6
C11—C6—C5	119.3 (9)	C18—C19—H19	119.6
C6—C7—C8	120.8 (11)	C19—C20—C21	120.7 (11)
C6—C7—H7	119.6	C19—C20—H20	119.6
C8—C7—H7	119.6	C21—C20—H20	119.6
C9—C8—C7	118.3 (12)	C20—C21—C22	120.4 (12)
C9—C8—H8	120.9	C20—C21—H21	119.8
C7—C8—H8	120.9	C22—C21—H21	119.8
C8—C9—C10	122.0 (12)	C17—C22—C21	119.5 (9)
C8—C9—H9	119.0	C17—C22—H22	120.3
C10—C9—H9	119.0	C21—C22—H22	120.3
			
N2—Pt1—N1—C5	51.4 (8)	C8—C9—C10—C11	−0.3 (19)
Br2—Pt1—N1—C5	−125.5 (7)	C9—C10—C11—C6	1.2 (16)
N2—Pt1—N1—C1	−116.7 (7)	C7—C6—C11—C10	−1.2 (15)
Br2—Pt1—N1—C1	66.4 (7)	C5—C6—C11—C10	179.0 (9)
N1—Pt1—N2—C16	55.9 (9)	C16—N2—C12—C13	2.7 (16)
Br1—Pt1—N2—C16	−118.6 (9)	Pt1—N2—C12—C13	174.1 (8)
N1—Pt1—N2—C12	−115.3 (8)	N2—C12—C13—C14	−4.8 (15)
Br1—Pt1—N2—C12	70.1 (8)	C12—C13—C14—C15	4.4 (15)
C5—N1—C1—C2	−1.1 (15)	C13—C14—C15—C16	−2.2 (14)
Pt1—N1—C1—C2	167.5 (8)	C12—N2—C16—C15	−0.2 (15)
N1—C1—C2—C3	−2.1 (16)	Pt1—N2—C16—C15	−170.9 (7)
C1—C2—C3—C4	3.6 (17)	C12—N2—C16—C17	−176.5 (9)
C2—C3—C4—C5	−2.0 (16)	Pt1—N2—C16—C17	12.9 (14)
C1—N1—C5—C4	2.7 (14)	C14—C15—C16—N2	0.1 (14)
Pt1—N1—C5—C4	−164.7 (7)	C14—C15—C16—C17	176.4 (9)
C1—N1—C5—C6	−174.7 (8)	N2—C16—C17—C22	45.9 (13)
Pt1—N1—C5—C6	17.9 (12)	C15—C16—C17—C22	−130.2 (9)
C3—C4—C5—N1	−1.2 (15)	N2—C16—C17—C18	−135.6 (10)
C3—C4—C5—C6	176.2 (9)	C15—C16—C17—C18	48.2 (13)
N1—C5—C6—C7	−132.2 (11)	C22—C17—C18—C19	−1.0 (14)
C4—C5—C6—C7	50.3 (13)	C16—C17—C18—C19	−179.4 (9)
N1—C5—C6—C11	47.6 (13)	C17—C18—C19—C20	−0.4 (16)
C4—C5—C6—C11	−129.9 (10)	C18—C19—C20—C21	1.5 (18)
C11—C6—C7—C8	0.2 (16)	C19—C20—C21—C22	−1.3 (17)
C5—C6—C7—C8	−180.0 (9)	C18—C17—C22—C21	1.2 (14)
C6—C7—C8—C9	0.7 (17)	C16—C17—C22—C21	179.7 (8)
C7—C8—C9—C10	−0.7 (18)	C20—C21—C22—C17	−0.1 (15)

## References

[bb1] Bruker (2000). *SADABS*, *SMART* and *SAINT* Bruker AXS Inc., Madison, Wisconsin, USA.

[bb2] Farrugia, L. J. (1997). *J. Appl. Cryst.* **30**, 565.

[bb3] Flack, H. D. (1983). *Acta Cryst.* A**39**, 876–881.

[bb4] Ha, K. (2011). *Z. Kristallogr. New Cryst. Struct.* **226**, 501–502.

[bb5] Ha, K. (2012). *Acta Cryst.* E**68**, m102. 10.1107/S1600536811055425 PMC3274849 22346796

[bb6] Sheldrick, G. M. (2008). *Acta Cryst.* A**64**, 112–122. 10.1107/S0108767307043930 18156677

[bb7] Spek, A. L. (2009). *Acta Cryst.* D**65**, 148–155. 10.1107/S090744490804362X PMC2631630 19171970

[bb8] Yoshinari, N., Kitani, N. & Konno, T. (2010). *Acta Cryst.* E**66**, m1499. 10.1107/S160053681004393X PMC3009048 21588912

